# Statins as a Therapeutic Approach for the Treatment of Pseudoxanthoma Elasticum Patients: Evaluation of the Spectrum Efficacy of Atorvastatin In Vitro

**DOI:** 10.3390/cells10020442

**Published:** 2021-02-19

**Authors:** Janina Tiemann, Christopher Lindenkamp, Ricarda Plümers, Isabel Faust, Cornelius Knabbe, Doris Hendig

**Affiliations:** Institut für Laboratoriums- und Transfusionsmedizin, Herz- und Diabeteszentrum Nordrhein-Westfalen, Universitätsklinik der Ruhr-Universität Bochum, 32545 Bad Oeynhausen, Germany; jtiemann@hdz-nrw.de (J.T.); clindenkamp@hdz-nrw.de (C.L.); rpluemers@hdz-nrw.de (R.P.); ifaust@hdz-nrw.de (I.F.); cknabbe@hdz-nrw.de (C.K.)

**Keywords:** pseudoxanthoma elasticum, atorvastatin, cholesterol biosynthesis

## Abstract

Pseudoxanthoma elasticum (PXE) is an autosomal recessive disorder caused by mutations in the *ATP-binding cassette sub-family C member 6* gene. Our previous studies revealed that PXE might be associated with premature aging. Treatment with statins showed positive effects not only for PXE but also for other diseases associated with premature aging like Hutchinson–Gilford progeria syndrome. Nevertheless, the molecular mechanisms in the case of PXE remain unclear. Thus, this study was performed to evaluate the efficiency of atorvastatin by analyzing key characteristics of the PXE phenotype in primary human dermal fibroblasts of PXE patients. Our data indicate that an atorvastatin treatment has a positive effect, especially on factors associated with cholesterol biosynthesis and prenylation processes, whereas the effect on age- and calcification-related factors was less pronounced.

## 1. Introduction

Pseudoxanthoma elasticum (PXE; OMIM #264800) is a genetic autosomal recessive disorder with a prevalence in the general population between 1:25000 and 1:100000. Characteristic clinical manifestations of PXE are small yellow papules which coalesce, leading to loose and wrinkled skin which appears on flexural regions like the neck and elbows and the development of angioid streaks in the retina which can bring on choroidal neovascularization and central vision loss [1,2,3]. Besides this, patients with PXE show cardiovascular changes which are characterized by mineralization and fragmentation of elastic fibers, an accumulation of proteoglycans and a higher prevalence of atherosclerotic plaques, resulting in a higher risk of atherosclerosis and coronary artery disease [4,5,6,7,8]. The crucial genetic cause of PXE is mutations in the *ATP-binding cassette sub-family C member 6* (ABCC6) gene, resulting in a deficiency of the encoded ABC transporter protein. ABCC6 is primarily expressed in the liver and to a small extent in peripheral tissues [9]. Molecular studies showed that a loss of function mutation of *ABCC6* results in a reduced circulating concentration of inorganic pyrophosphate (PPi), which is known as a calcification inhibitor [10,11,12]. The mechanism connecting an *ABCC6* deficiency to the clinical and molecular manifestations of PXE is not completely understood. Furthermore, the physiological substrate of the ABCC6 transporter is still unknown, making the development of a specific therapy difficult.

Besides this, studies showed molecular characteristic similarities between PXE and other heritable diseases like Hutchinson–Gilford progeria syndrome (HGPS). Both diseases are characterized by decreased adenosine triphosphate (ATP) and PPi plasma levels, an increased alkaline phosphatase activity and disrupted PPi homeostasis [13,14,15]. In addition, HGPS pathogenesis clearly shows characteristic features of accelerated aging [16]. A previous study also showed an association between PXE and premature aging, with dysregulation in *growth differentation factor 11* and *insulin-like growth factor binding protein 3* expression, which potentially play a role in senescence-associated secretory phenotype (SASP) [17].

It was further shown that the progression of HGPS could be slowed down by combined therapy with bisphosphonates and statins [18]. For PXE, a therapy with statins was also shown to have a positive effect on disease progression [19,20]. Because of the unknown substrate of ABCC6 and the incomplete understanding of the molecular pathomechanism of PXE, the effect of statins on a molecular level remains unknown. In general, statins are potent inhibitors of cholesterol biosynthesis, but also show anti-inflammatory effects and can significantly reduce C-reactive protein in serum [21,22]. In cholesterol biosynthesis, statins inhibit 3-hydroxy-3-methylglutaryl coenzyme A (HMG-CoA), which converts HMG to L-mevalonate. Thus, statins directly intervene in lipid metabolism and indirectly in the protein prenylation process, which take place downstream of the cholesterol biosynthesis pathway [21,23,24]. Kuzaj et al. found dysregulations in the lipid metabolism of primary human dermal fibroblasts of PXE patients, as there is increased activity of HMG-COA reductase, strongly elevated transcript and protein levels of proprotein convertase subtilisin/kexin type 9 (PCSK9) and a significant reduction in *APOE* mRNA expression, which probably give a first explanation for the positive effect of statins on PXE pathogenesis. Furthermore, *ABCC6* expression in healthy fibroblasts strongly increased under lipoprotein-deficient conditions, which suggest a functional role of ABCC6 in lipid homeostasis [25].

As mentioned before, statin therapy had positive effects in PXE and, in particular, treatment with atorvastatin (AT) seems to counteract tissue calcification in *Abcc6^−/−^* mice [19]. An extended analysis of AT treatment on a molecular level may better explain the effect on PXE and help to understand the molecular pathomechanism. Thus, this is the first study evaluating the influence of AT on different known dysregulated factors in primary human dermal fibroblasts of PXE patients in vitro. Through this, we were able to give further insights into the effects of AT on a molecular level in peripheral tissues.

## 2. Materials and Methods

### 2.1. Experimental Design

The study was designed to evaluate the effect and the spectrum of efficacy of AT on primary human dermal fibroblasts of PXE patients, as previous studies showed that cholesterol biosynthesis seems to play a crucial role in PXE pathogenesis. Thus, PXE fibroblasts and fibroblasts from healthy controls were cultivated in medium with 10% lipoprotein-deficient fetal calf serum (LPDS), to trigger the PXE phenotype, and, thus, facilitate the evaluation of potential effects of AT on PXE fibroblasts. The effect of AT and its spectrum of efficacy was determined by measuring the relative mRNA expression of factors directly involved in cholesterol biosynthesis, like *3-hydroxy-3-methyl-glutaryl-coenzyme A (HMGCR)*, *farnesyl pyrophosphate synthase* (*FDPS)*, *low density lipoprotein receptor* (*LDLR)* and *high density lipoprotein binding protein (HDLBP)*, as well as of factors which are, through their association with prenylation processes, indirectly connected to cholesterol metabolism, like *Lamin B1 (LMNB1)*, *zinc metallopeptidase STE 24 (ZMPSTE24)* and prenyl-cysteine Oxidase 1 (*PCYOX)*. Because it is known that statins have an anti-inflammatory effect as well as an influence on calcification processes, gene expression and protein concentration in cell culture supernatants of secreted factors associated with a proinflammatory senescence-associated secretory phenotype, such as interleukin-6 (IL6), insulin-like growth factor-binding protein 3 (IGFBP3) and growth differentiation factor (GDF11), as well as gene expression of the anti-calcification factors *ectonucleotide pyrophosphatase/phosphodiesterase 1 (ENPP1)* and *osteopontin (OPN)*, were determined.

### 2.2. Cell Culture

Normal human dermal fibroblasts (NHDFs) were purchased from the Coriell Institute for Medical Research (Camden, NJ, USA). Primary human dermal fibroblasts from PXE patients were isolated from skin biopsies. Fibroblast characteristics are listed in Table 1. All patients and controls gave their informed consent for using the material for research purposes. The study was conducted in accordance with the Declaration of Helsinki and approved by the Ethics Committee of the HDZ NRW, Department of Medicine, Ruhr-University of Bochum (registry no. 32/2008, approval date is 3rd November 2008).

NHDFs as well as PXE fibroblasts were cultivated in Dulbecco’s modified essential medium (DMEM, Gibco, Thermo Fisher Scientific, San Diego, CA, USA) supplemented with 10% fetal calf serum (FCS) (Biowest, Aidenbach, Germany), 2% L-glutamine (200 mM) (PAN Biotech, Eidenach, Germany) and 1% antibiotic/antimycotic solution (PAA Laboratories, Pasching, Austria). Fibroblasts were subcultured after reaching confluency.

In this study, fibroblasts between passage 9 and 12 were used and triplicates were prepared for every biological sample. For all experiments, NHDF and PXE fibroblasts were seeded in a final cell density of 177 cells/mm^2^ and cultivated for 24 h in medium supplemented with 10% FCS. Afterwards, the medium was changed to medium supplemented with 10% LPDS or medium supplemented with 10% LPDS and 20 µM AT for an additional 72 h.

### 2.3. Delipidation of FCS

LPDS was prepared according to Gibson et al. [26]. In brief, 50 mL FCS were incubated with 1 g Cab-o-sil (silicic acid powder, Sigma, Taufkirchen, Germany) at 4 °C overnight. The next day, the mixture was centrifuged at 5000× *g* for 1 h at 4 °C. The clarified supernatant was transferred to a new tube and stored at −20 °C until further use. Before the preparation of fresh delipidated medium, LPDS was sterile filtered (0.2 µm). Through delipidation, the concentration of free cholesterol was lowered by about 78%, low density lipoprotein (LDL) by about 95% and high density lipoprotein (HDL) by about 57%, whereas triglyceride concentrations remained unchanged.

### 2.4. Nucleic Acid Isolation

A NucleoSpin RNA Kit (Macherey-Nagel, Düren, Germany) was used for RNA isolation. DNA isolation was done using a NucleoSpin Blood Extraction Kit (Macherey-Nagel, Düren, Germany). DNA content was used for normalization of immunoassay measurements. RNA and DNA isolation was performed according to the manufacturer’s instructions.

### 2.5. Gene Expression Analysis

SuperScript II Reverse Transcriptase (Thermo Fisher Scientific, San Diego, CA, USA) was used to transcribe 1 µg RNA into cDNA. For all gene expression measurements, 2.5 μL cDNA (1:10), 0.25 μL forward and reverse primer (Biomers, Ulm, Germany) with a final concentration of 25 µM (except IL6: 20 µM), 2.0 μL water and 5.0 μL LightCycler 480 SYBR Green I Master reaction mixture (Roche, Penzberg, Germany) were mixed. The qPCR protocol involves an initial incubation for 5 min at 95 °C and 45 subsequent cycles of denaturation (95 °C, 10 s), annealing (specific annealing temperature, 15 s) and elongation (72 °C, 20 s). *β-actin (ACTB), glycerinaldehyde-3-phosphate-dehydrogenase (GAPDH)* and *β2-microglobulin (β2M)* mRNA expression was used for normalization. Melting curve analysis was done after amplification and technical triplicates were performed for each biological sample. Measurements were carried out using a LightCycler 480 (Roche, Penzberg, Germany). Relative mRNA expression was calculated using the delta delta Ct method considering PCR efficiency. Sequences of the primers used for qPCR are listed in Table 2.

### 2.6. Immunoassays for Evaluation of SASP Factors in Cell Culture Supernatants

Protein concentrations of IL6 in cell culture supernatants were determined using the immunoanalyzer Cobas e411 (Roche, Basel, Switzerland). Measurements of IGFBP3 concentrations in cell culture supernatants were conducted using a commercially available ELISA kit (R&D Systems, Abingdon, UK). The results of both measurements were normalized to DNA content.

### 2.7. Evaluation of ENPP1 Activity in Cell Culture Supernatants

ENPP1 activity in cell culture supernatants was determined as described before [27]. As the substrate of ENPP1, tyhmidin-5′-monophosphate-*p*-nitrophenyl ester sodium salt (Sigma-Aldrich, St. Louis, MO, USA) (1 mg/mL) was used. The substrate was added to media and fibroblasts were cultivated for 1 h at 37 °C and 5% CO_2_. After incubation, the formed *p*-nitrophenol was measured at 415 nm using a *Tecan infinite m200pro* (Tecan, Switzerland). Results were normalized to DNA content.

### 2.8. Statistical Analysis

Data are shown as mean ± standard error (SEM). GraphPad Prism 5.0 was used as statistical software. The non-parametric two-tailed Mann–Whitney U test was performed for statistical analyses. *p*-values of 0.05 or less were considered statistically significant.

## 3. Results

### 3.1. Atorvastatin Has Positive Effects on Gene Expression of Targets Linked to Cholesterol Biosynthesis

**Figure 1:**
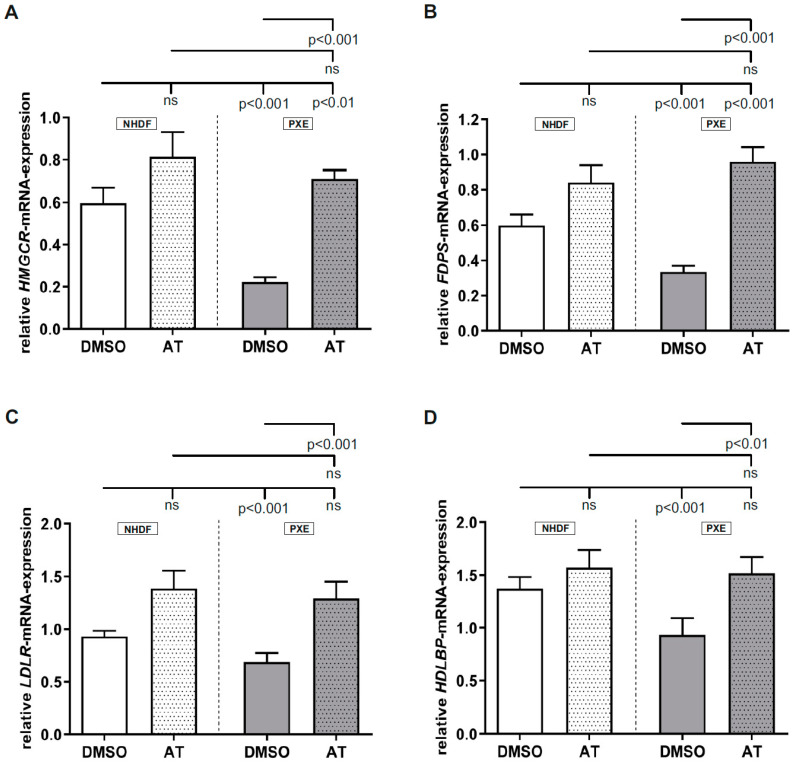
Relative *HGMCR, FDPS*, *LDLR* and *HDLBP* mRNA expression of Pseudoxanthoma elasticum (PXE) fibroblasts and Normal human dermal fibroblasts (NHDFs). Fibroblasts were cultivated for 72 h in medium with 10% lipoprotein-deficient fetal calf serum (LPDS) or medium with 10%LPDS and 20 µM atorvastatin (AT). (**A**) Relative *HMGCR* mRNA expression of PXE fibroblasts (gray, *n* = 3) and NHDFs (white, *n* = 3). (**B**) Relative *FDPS* mRNA expression of PXE fibroblasts (gray, *n* = 3) and NHDFs (white, *n* = 3). (**C**) Relative *LDLR* mRNA expression of PXE fibroblasts (gray, *n* = 3) and NHDFs (white, *n* = 3). (**D**) Relative *HDLBP* mRNA expression of PXE fibroblasts (gray, *n* = 3) and NHDFs (white, *n* = 3). Data are shown as mean ± SEM. ns: not significant, *p* > 0.05.

Determination of *HMGCR*, *FDPS*, *LDLR* and *HDLBP* mRNA expression (Figure 1A–D) showed no significant changes between AT-treated samples and DMSO-treated controls. For all analyzed targets, a significant decrease in gene expression was observed for DMSO-treated PXE fibroblasts compared to DMSO-treated NHDFs (*HMGCR*, NHDF DMSO: 0.60 ± 0.07, PXE DMSO: 0.22 ± 0.02; *FDPS*, NHDF DMSO: 0.60 ± 0.06, PXE DMSO: 0.34 ±0.03; *LDLR*, NHDF DMSO: 0.93 ± 0.05; PXE DMSO: 0.67 ± 0.08; *HDLBP*, control DMSO: 1.37 ± 0.11; PXE DMSO: 0.93 ± 0.16.). Upon application of 20 µM AT, all targets showed a significant increase in mRNA expression in PXE fibroblasts compared to the DMSO-treated PXE fibroblasts, Figure 1A–D: *HMGCR*; PXE DMSO: 0.22 ± 0.02, PXE AT: 0.71 ± 0.04; *FDPS*; PXE DMSO: 0.34 ± 0.03, PXE AT: 0.96 ±0.08, *LDLR*; PXE DMSO: 0.69 ± 0.08; PXE AT: 1.29 ± 0.16, *HDLBP*: PXE DMSO: 0.93 ± 0.16; PXE AT: 1.52 ± 0.15. This leads to a nearly equal gene expression for targets linked to cholesterol biosynthesis in the AT-treated PXE fibroblasts compared to AT-treated NHDFs. In case of *LDLR* and *HDLBP* gene expression, PXE fibroblasts showed a comparable expression even to vehicle-treated NHDFs when treated with 20 µm AT. For *HMGCR* and *FDPS* mRNA expression, a significant induction of gene expression was observed for PXE fibroblasts when treated with 20 µM AT compared to DMSO-treated NHDFs (HMGCR; NHDF DMSO: 0.60 ± 0.07; PXE AT: 0.71 ± 0.04, FDPS; 0.6 ± 0.06; PXE AT: 0.96 ± 0.08).

### 3.2. Atorvastatin Has Positive Effects on Gene Expression of Factors Involved in Prenylation Processes

**Figure 2:**
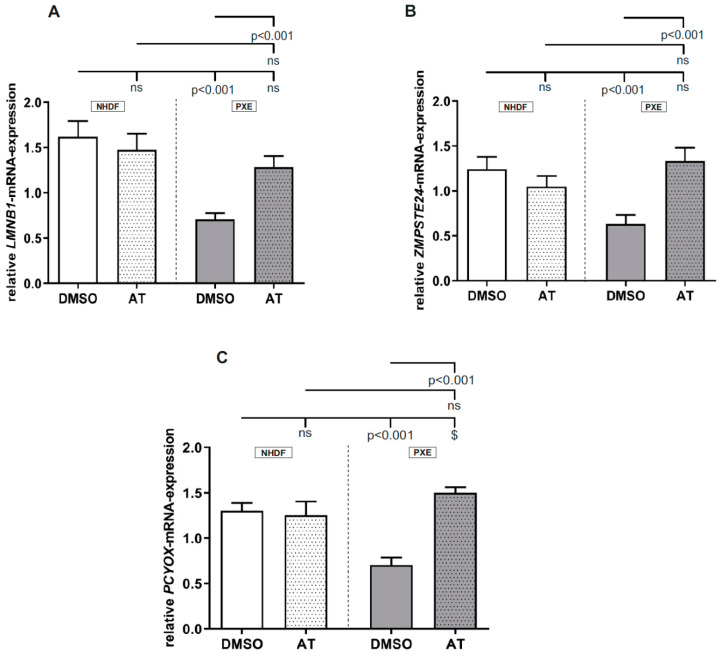
Relative *LMNB1*, *ZMPSTE24* and *PCYOX* mRNA expression of PXE fibroblasts and NHDFs. Fibroblasts were cultivated for 72 h in 10% LPDS medium or medium with 10% LPDS and 20 µM AT. (**A**) Relative *LMNB1* mRNA expression PXE fibroblasts (gray, *n* = 3) and NHDFs (white, *n* = 3). (**B**) Relative *ZMPSTE24* mRNA expression of PXE fibroblasts (gray, *n* = 3) and NHDFs (white, *n* = 3). (**C**) Relative *PCYOX* mRNA expression of PXE fibroblasts (gray, *n* = 3) and NHDFs (white, *n* = 3).

As seen in Figure 2A–C, no significant differences were observed between DMSO-treated controls and AT-treated controls for all measured targets. However, all measured targets showed a significant decrease in mRNA expression for DMSO-treated PXE fibroblasts compared to DMSO-treated NHDFs (*LMNB1*, NHDF DMSO: 1.62 ± 0.18, PXE DMSO: 0.71 ± 0.07; *ZMPSTE24*, NHDF DMSO: 1.24 ± 0.14, PXE DMSO: 0.63 ±0.10; *PCYOX,* NHDF DMSO: 1.30 ± 0.09; PXE DMSO: 0.70 ± 0.08). When treated with 20 µM AT, a significant increase in mRNA expression of all measured targets was observed for PXE fibroblasts compared to DMSO-treated PXE fibroblasts (*LMNB1*, PXE DMSO: 0.71 ± 0.07; PXE AT: 1.29 ± 0.12; *ZMPSTE24*, PXE DMSO: 0.63 ± 0.10, PXE AT: 1.33 ± 0.15; *PCYOX*, PXE DMSO: 0.70 ± 0.08; PXE AT: 1.50 ± 0.06). The increase in mRNA expression in PXE fibroblasts when treated with AT leads to nearly equal gene expression for all analyzed targets compared to the gene expression of AT-treated NHDFs. We observed significant differences for *LMNB1* and ZMPSTE24 gene expression in PXE fibroblasts when compared to DMSO-treated NHDFs. For *PCYOX*, a significant increase in gene expression was determined for PXE fibroblasts compared to DMSO-treated NHDFs (NHDF DMSO: 1.30 ± 0.09; PXE DMSO: 1.60 ± 0.06). 

### 3.3. Limited Effect of Atorvastatin on Inflammatory Markers

**Figure 3:**
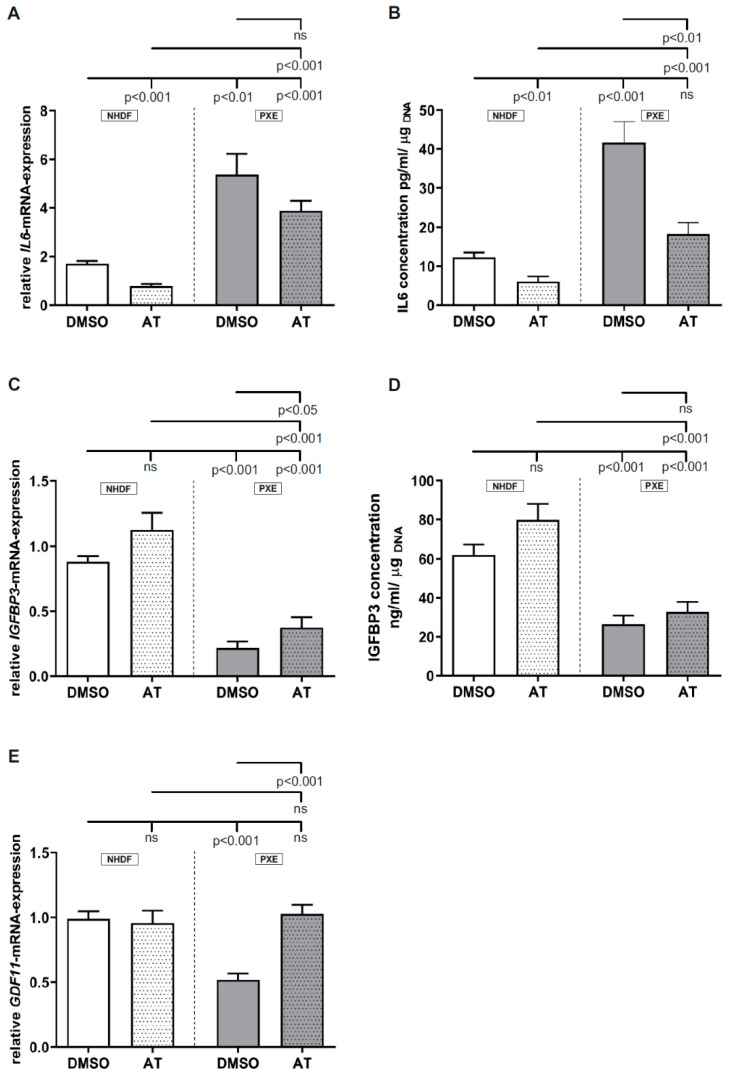
Relative *IL6*, *IGFBP3* and GDF11 mRNA expression as well as IL6 and IGFBP3 protein concentration in cell culture supernatants of PXE fibroblasts and NHDFs. Fibroblasts were cultivated for 72 h in medium with 10% LPDS or medium with 10% LPDS and 20 µM AT. (**A**) Relative *IL6* mRNA expression of PXE fibroblasts (gray, *n* = 3) and NHDFs (white, *n* = 3). (**B**) IL6 protein concentration in cell culture supernatants of PXE fibroblasts (gray, *n* = 3) and NHDFs (white, *n* = 3). (**C**) Relative *IGFBP3* mRNA expression of PXE fibroblasts (gray, *n* = 3) and NHDFs (white, *n* = 3). (**D**) IGFBP3 protein concentration in cell culture supernatants of PXE fibroblasts (gray, *n* = 3) and NHDFs (white, *n* = 3). (**E**) Relative *GDF11* mRNA expression of PXE fibroblasts (gray, *n* = 3) and NHDFs (white, *n* = 3. Data are shown as mean ± SEM. ns: not significant, *p* > 0.05.

Measurement of *IL6* gene expression (Figure 3A; NHDF DMSO: 1.71 ± 0.11; PXE DMSO: 5.37 ± 0.86) as well as protein concentration in cell culture supernatants (Figure 3B; NHDF DMSO: 12.3 ± 1.17 pg/mL; PXE DMSO: 41.6 ± 5.42 pg/mL) showed a significant increase in the case of DMSO-treated PXE fibroblasts compared to DMSO-treated NHDFs. On application of 20 µM AT, a significant decrease in *IL6* mRNA expression could be observed for NHDFs compared to the vehicle-treated NHDFs (NHDF DMSO: 1.71 ± 0.11; NHDF AT: 0.79 ± 0.08). PXE fibroblasts treated with 20 µM AT also showed a decrease in *IL6* gene expression, which did not reach statistical significance. For IL6 protein concentration in cell culture supernatants, a significant decrease was observed for AT-treated NHDFs (NHDF DMSO: 12.33 ± 1.17 pg/mL; NHDF AT: 6.10 ± 1.27 pg/mL) as well as PXE fibroblasts (PXE DMSO: 41.64 ± 5.42 pg/mL; PXE AT: 18.33 ± 2.92 pg/mL) compared to the DMSO-treated NHDFs. AT treatment of PXE fibroblasts did not lead to equal *IL6* mRNA expression or protein concentration in cell culture supernatants compared to AT-treated NHDFs. Thus, AT-treated PXE fibroblasts still showed a significantly increased *IL6* gene expression (NHDF AT: 0.79 ± 0.08; PXE AT: 3.89 ± 0.40) as well as protein concentration in cell culture supernatants (NHDF AT: 6.10 ± 1.27 pg/mL; PXE AT: 18.33 ± 2.92 pg/mL) compared to AT-treated NHDFs. Compared to the DMSO-treated NHDFs, *IL6* gene expression in AT-treated PXE fibroblasts was significantly increased (control DMSO: 1.71 ± 0.11; PXE AT: 3.89 ± 0.40), whereas IL6 protein concentration in cell culture supernatants of AT-treated PXE fibroblasts showed no differences compared to DMSO-treated NHDFs. 

Determination of *IGFBP3* mRNA expression (Figure 3C; NHDF DMSO: 0.88 ± 0.04; PXE DMSO: 0.22 ± 0.05) and protein concentration in cell culture supernatants (Figure 3D; NHDF DMSO: 62.16 ± 5.27 ng/mL; PXE DMSO: 26.58 ± 4.41 ng/mL) showed a significant decrease for DMSO-treated PXE fibroblasts compared to DMSO-treated NHDFs. When treated with 20 µM AT, an increase in *IGFBP3* gene expression as well as protein concentration in cell culture supernatants could be observed for NHDFs as well as PXE fibroblasts compared to the respective controls. However, this only reached statistical significance in the case of *IGFBP3* gene expression in AT-treated PXE fibroblasts compared to DMSO-treated PXE fibroblasts (PXE DMSO: 0.22 ± 0.05; PXE AT: 0.38 ± 0.08). Compared to the AT-treated and DMSO-treated NHDFs, AT-treated PXE fibroblasts still showed a significantly decreased *IGFBP3* gene expression: NHDF DMSO: 0.88 ± 0.04; NHDF AT: 1.13 ± 0.13; PXE AT: 0.38 ± 0.08. Protein concentrations in cell culture supernatants were also still decreased in PXE fibroblasts: NHDF DMSO: 62.16 ± 5.27 ng/mL; NHDF AT: 79.91 ± 8.23 ng/mL; PXE AT: 32.91 ± 5.04 ng/mL).

As seen in Figure 3E, the measurement of *GDF11* gene expression showed a significant decrease for DMSO-treated PXE fibroblasts compared to DMSO-treated NHDFs (NHDF DMSO: 0.99 ± 0.06; PXE DMSO: 0.52 ± 0.05). When treated with 20 µM AT, no differences in *GDF11* gene expression could be observed for NHDFs compared to DMSO-treated NHDFs but a significant increase in *GDF11* gene expression could be detected for AT-treated PXE fibroblasts compared to DMSO-treated PXE fibroblasts (PXE DMSO: 0.52 ± 0.05; PXE AT: 1.03 ± 0.07). No differences for *GDF11* gene expression were seen between AT-treated PXE fibroblasts and AT-treated NHDFs as well as between AT-treated PXE fibroblasts and DMSO-treated NHDFs.

### 3.4. Limited Effect of Atorvastatin on Gene Expression of Calcification-Associated Factors

**Figure 4:**
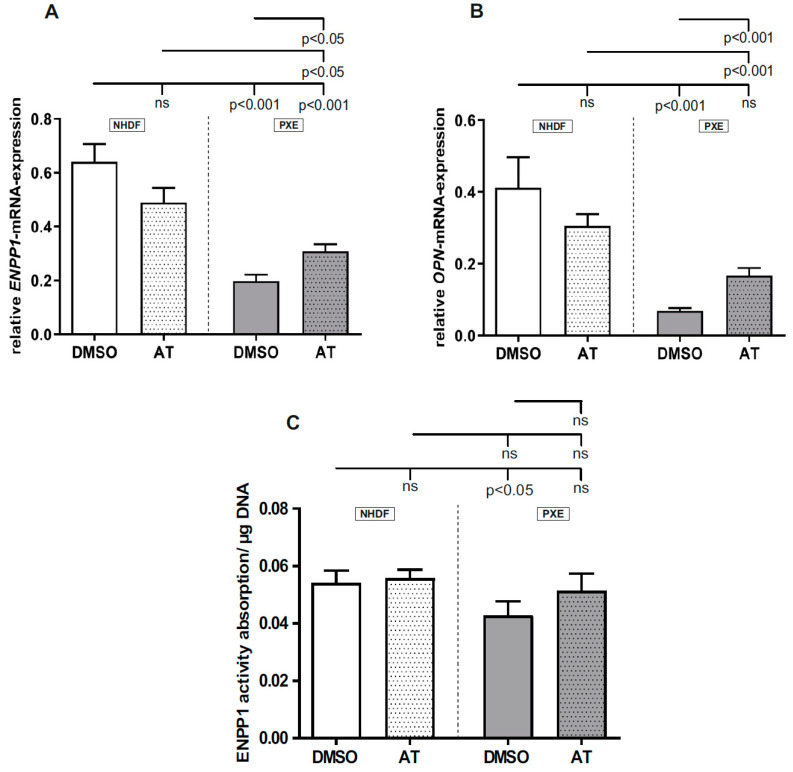
Relative *ENPP1* and *OPN* mRNA expression as well as ENPP1 activity of PXE fibroblasts and NHDFs. Fibroblasts were cultivated for 72 h in medium with 10% LPDS or medium with 10% LPDS and 20 µM AT. (**A**) Relative *ENPP1* mRNA expression PXE fibroblasts (gray, *n* = 3) and NHDFs (white, *n* = 3). (**B**) Relative *OPN* mRNA expression PXE fibroblasts (gray, *n* = 3) and NHDFs (white, *n* = 3). (**C**) ENPP1 activity of PXE fibroblasts (gray, *n* = 3) and NHDFs (white, *n* = 3). Data are shown as mean ± SEM. ns: not significant, *p* > 0.05.

Analysis of *ENPP1* (NHDF DMSO: 0.64 ± 0.07; PXE DMSO: 0.20 ± 0.02) and *OPN* (NHDF DMSO: 0.41 ± 0.08; PXE DMSO: 0.07 ± 0.01) gene expression and ENPP1 activity (NHDF DMSO: 0.0541 ± 0.0043; PXE DMSO 0.043 ± 0.0049) showed a significant decrease for DMSO-treated PXE fibroblasts compared to DMSO-treated NHDFs (Figure 4A–C). On application of 20 µM AT, no significant differences in *ENPP1* and *OPN* mRNA expression and ENPP1 activity were observed for NHDFs compared to DMSO-treated NHDFs but there was a significant increase for PXE fibroblasts compared to DMSO-treated PXE fibroblasts for both targets on the mRNA level (*ENPP1*, PXE DMSO: 0.20 ± 0.02; PXE AT: 0.31 ± 0.03; *OPN*, PXE DMSO: 0.07 ± 0.01; PXE AT: 0.17 ± 0.02). Additionally, no significant effect of 20 µM AT was shown on ENPP1 activity of PXE fibroblasts compared with DMSO-treated PXE fibroblasts. However, AT-treated PXE fibroblasts still showed a significantly decreased *ENPP1* (NHDF DMSO: 0.64 ± 0.07; control AT: 0.50 ± 0.05; PXE AT: 0.31 ± 0.03) and *OPN* (NHDF DMSO: 0.41 ± 0.08; NHDF AT: 0.31 ± 0.03; PXE AT: 0.17 ± 0.02) gene expression compared to AT-treated as well as DMSO-treated NHDFs.

## 4. Discussion

PXE is mainly caused by mutations in the *ABCC6* gene which leads to a deficiency of the appropriate ABC transporter protein. Due to the fact that the physiological substrate of the ABCC6 transporter and, thus, the pathomechanism of PXE is still unknown, there are no specific therapies for an effective treatment for PXE patients that are available. However, a survey of 1747 PXE patients showed that one third of these patients currently take or have taken cholesterol-lowering drugs in the past [1]. The reason why the administration of statins leads to a positive effect in PXE patients remains mostly unknown, although our previous studies showed a possible association of PXE with the alteration of cholesterol biosynthesis [25]. Thus, in a first step, we evaluated the effect of AT on the gene expression of factors directly associated with cholesterol biosynthesis. For *HMGCR*, *LDLR*, *FDPS* and *HDLBP* mRNA expression, a significant decrease could be observed for DMSO-treated PXE fibroblasts compared to DMSO-treated NHDFs. HMGCR and LDLR play an essential role in the regulation of cholesterol homeostasis, as cholesterol can either be synthesized from acetyl-CoA and mevalonate by HMGCR or it can be taken up by LDL and LDLR. By the mechanism of end-product repression, HMGCR activity, as well as LDLR expression, are reduced to avoid cholesterol overload [28]. A study of Kuzaj et al. showed a significant induction of HMGCR activity in PXE fibroblasts compared to NHDFs after 24 h of cultivation [25]. However, we observed a reduction of *HMGCR* and *LDLR* gene expression in PXE fibroblasts after 72 h of cultivation, which could be a direct compensatory result of induced HMGCR activity. As FDPS is also directly associated with cholesterol biosynthesis through its function to synthesize the intermediate farnesylpyrophosphate, the downregulation of the *FDPS* gene expression in PXE fibroblasts might also be a direct mechanism to avoid cholesterol overload or an overload of the intermediate itself. In contrast to the other targets, the role of HDLBP in cholesterol biosynthesis is still not clear. However, a study of McKnight et al. showed a correlation between an intracellular accumulation of cholesterol and subsequently increased *HDLBP* mRNA expression in J774 macrophages [29]. McKnight et al. demonstrated further that an overexpression of HDLBP in mammalian cells leads to an increased binding of HDL to the cell surface, possibly facilitating cholesterol efflux [29]. Additionally, Chiu et al. showed that HDLBP is expressed in arteriosclerotic plaques [30]. According to these studies, the decreased *HDLBP* gene expression seen here would, thus, contradict the hypothesis of an increased intracellular cholesterol content in PXE fibroblasts. Nevertheless, a previous study of Kuzaj et al. showed a decreased *HDLBP* gene expression for PXE fibroblasts compared to controls [25], supporting the results of this study. The strong association of HDLBP as well as of HMGCR, LDLR and FDPS with the cholesterol biosynthetic pathway is underlined by the fact that upon application of AT in PXE, the gene expression of all targets could be significantly raised to the expression level of the NHDFs. The AT treatment could, thus, probably reduce HMGCR activity, accompanied by an increase in the gene expression of *LDLR* and *FDPS*, leading to a normalization of cellular cholesterol levels.

We further analyzed the gene expression of *LMNB1*, *ZMPSTE24* and *PCYOX*, which also have a strong association with cholesterol biosynthesis, and especially with HMGCR, as they play a crucial role in prenylation processes. Thus, Lacher et al. demonstrated that HMGCR-deficient T cells can be rescued through an exogenous application of geranlygeranylpyrophosphate, another intermediate of the cholesterol biosynthetic pathway and a substrate for the prenylation of different proteins. Moreover, it was independent of cholesterol content as the exogenous application of cholesterol had no effect on these cells [31]. For all three analyzed targets, a significant decrease in gene expression could be observed for DMSO-treated PXE fibroblasts compared to DMSO-treated NHDFs. Because of the close association of prenylation processes with HMGCR, the observed induction of HMGCR activity in PXE fibroblasts could, therefore, lead to a reduced gene expression of *LMNB1* and *ZMPSTE24*, as well as *PCYOX*, to guarantee protein homeostasis of prenylated proteins. On application of AT, a similar effect, as seen before for *HMGCR*, *LDLR*, *FDPS* and *HDLBP*, can be observed. Thus, the gene expression of all three targets was almost raised to the gene expression levels of DMSO- and AT-treated NHDFs. This further strengthens the assumption that *LMNB1*, *ZMPSTE24* and *PCYOX* mRNA expression is closely associated with HMGCR activity to control protein homeostasis. It further shows that an AT treatment has a direct effect on prenylation processes. 

As a previous study showed that PXE might be associated with premature aging processes [17], we additionally evaluated the gene expression of *IL6*, *IGFBP3* and *GDF11*, which play a pivotal role in the senescence-associated secretory phenotype (SASP) and were partly shown to be dysregulated in PXE [17]. A study further showed that AT can reduce IL6 production in human endothelial cells in a concentration-dependent manner [32]. We detected an increase in IL6 gene expression and protein concentration for DMSO-treated PXE fibroblasts compared to DMSO-treated controls, pointing towards a proinflammatory secretory phenotype for PXE fibroblasts. The application of AT slightly reduced IL6 gene expression and significantly reduced IL6 protein concentration. However, a total normalization of IL6 levels of AT-treated PXE fibroblasts and DMSO- and AT-treated NHDFs was not achieved. The known anti-inflammatory effect of statins is often traced back to the inhibition of the prenylation of small GTPases and thus the inhibition of the JAK-STAT signal pathway [33]. The fact that we could not achieve a total normalization of IL6 levels through the AT treatment does not exclude the involvement of the JAK-STAT signal pathway but might indicate the need for higher AT concentrations or the relevance of other potentially age-associated pathways. In the case of IGFBP3, we could observe a reduction of gene expression as well as protein concentration for DMSO-treated PXE fibroblasts compared to DMSO-treated NHDFs, which confirms our previous results [17], indicating a potential dysregulation of IGF1 signaling in peripheral tissues of PXE patients. Similar to IL6, we could not achieve a normalization of IGFBP3 gene expression and protein concentration for PXE fibroblasts when treated with AT. A study of Narayanan et al. showed a reduction of circulating IGFBP3 in patients with type 2 diabetes when treated with AT [34]. However, it is known that tissue-specific expression can differ from circulating IGFBP3 concentrations [35] which can, thus, cause divergent results in peripheral tissues of PXE patients. Nevertheless, the effect of AT on IGFBP3 gene expression and protein concentration seems to be limited. For *GDF11* gene expression, a reduction of gene expression was shown for DMSO-treated PXE fibroblasts compared to DMSO-treated controls, which once more confirms previous results [17], even though different cultivation conditions were applied. In contrast to IL6 and IGFBP3, the application of AT led to the normalization of *GDF11* gene expression in PXE fibroblasts compared to DMSO- and AT-treated controls, which might indicate an association between *GDF11* gene expression and HMGCR activity. Additionally, a previous study demonstrated that GDF11 can reduce IL6 expression in *apoE*^−/−^ mice as well as the levels of proinflammatory cytokines in macrophages [36], which points towards an anti-inflammatory effect of GDF11.

Besides the anti-inflammatory effect of AT, previous studies showed that a treatment with AT can also have an inhibitory effect on calcification processes. Thus, Guo et al. demonstrated that an AT treatment can inhibit further mineralization in *Abcc6*^−/−^ mice, although the molecular mechanisms remained unclear [19]. However, it is known that ENPP1 expression and activity, which are closely associated with calcification processes, are reduced in PXE [37]. ENPP1 plays a role in the hydrolysis of nucleotide triphosphates and the generation of PPi, which is a potent calcification inhibitor [18,38,39,40]. We could confirm the previous results of dysregulated reduced *ENPP1* gene expression and ENPP1 activity in PXE fibroblasts compared to controls but the effect of AT on *ENPP1* gene expression as well as ENPP1 activity was limited. ENPP1 is further associated with OPN gene expression [38], another potent calcification inhibitor [41,42]. We showed reduced OPN expression for DMSO-treated PXE fibroblasts compared to DMSO-treated NHDFs, which potentially contributed to the mineralization seen for PXE. On application of AT, we detected a significant increase in *OPN* gene expression in PXE fibroblasts compared to DMSO-treated PXE fibroblasts. This increase did not lead to a normalization of *OPN* expression in AT-treated PXE fibroblasts compared to DMSO- and AT-treated NHDFs. This further strengthens the assumption of a limited effect of AT on the studied anticalcification factors.

In conclusion, this is the first study analyzing the spectrum efficacy of an AT treatment of primary human dermal fibroblasts of PXE patients on the molecular level. Our data indicate that a treatment with AT primarily effects factors directly associated with the cholesterol biosynthetic pathway and prenylation processes. We point out that the protein expression of some of investigated genes was not analyzed due to a limited availability of good and specific antibodies. Therefore, protein expression may differ from mRNA expression.

However, the effect of AT on age- and calcification-related factors in PXE seems to be limited. Thus, further studies, combining statin and bisphosphonate and/or anti-inflammatory treatment in vitro, will be of great interest to evaluate their effect on the molecular mechanism of the PXE phenotype and could help in better understanding the molecular pathomechanism.

## Figures and Tables

**Table 1:** Characteristics of fibroblasts used.

Sample ID	Gender	Age ^1^	Biopsy Source	*ABCC6* Genotype ^2^		Genotype Status	Phenodex Score ^3^
**PXE Patients**							
**P3M ^a^**	Male	57	Neck	c.3421C > T (p.(Arg1141*)	c.3883 − 6G > A (SSM)	cht	S3, V2; C0
**P128M ^a^**	Male	51	Neck	c.3769_3770insC (p.L1259fsX1277)	c.3769_3770insC (p.L1259fsX1277)	hm	S2; E2; G0; C1
**P255F ^a^**	Female	48	Arm	c.3421C > T (p.Arg1141*)	c.2787 + 1G > T	cht	S3; E2; G0; C0
**Healthy Controls**							
**M57A ^b^** (**AG13145**)	Male	57	Arm	-	-	wt	Not applicable
**M52A ^b^** (**AG11482**)	Male	52	Arm	-	-	wt	Not applicable
**F48A ^b^** (**AG14284**)	Female	48	Arm	-	-	wt	Not applicable

hm, homozygous; cht, compound heterozygous; wt, wild type; SSM, splice site mutation. ^a^ Fibroblasts isolated from skin biopsies. ^b^ Fibroblasts purchased from Coriell Institute for Medical Research (Camden, NJ, USA). ^1^ Age in years. ^2^ Nucleotide numbering refers to the cDNA sequence with the A of the ATG translation initiation start site as nucleotide +1 (GenBank accession number NM_001171.2). ^3^ Adapted from the Phenodex score (an internationally standardized scoring system for uniform evaluation of PXE cases) according to Legrand et al. [10]. S: skin; E: eye; G: gastrointestinal; V: vascular; C: cardiac.

**Table 2:** Primer sequences used for quantitative real-time PCR.

Gene	Protein	5′-3′ Sequence	Reference ^1^	Annealing Temperature (°C)	Efficiency
*β-ACTIN* *Beta-actin*	ß-Actin	CGCGAGAAGATGACCC ATTGCCAATGGTGATGAC	NM_001101	59	2.0
*GAPDH* *Glycerinaldehyd-3-phosphat-dehydrogenase*	GAPDH	AGGTCGGAGTCAACGGAT TCCTGGAAGATGGTGATG	NM_002046	59	1.8
*β2M* *Beta-2-* *microglobulin*	ß2M	TGTGCTCGCGCTACTCTCTCTT CGGATGGATGAAACCCAGACA	NM_004048	59	2.0
*ENPP1* *Ectonucleotide pyrophosphatase/phosphodiesterase 1*	ENPP1	AATGCCCCTTTGGACACT CCCGTAACTTTTGGT	NM 006,208	59	1.8
*IL6* *Interleukin 6*	IL6	ACAGCCACTCACCTCTTCAG GTGCCTCTTTGCTGCTTTCAC	NM 000600.4	63	1.9
*LMNB1* *Lamin B1*	Lamin B1	GCAGACTTACCATGCCAAAC TCCCTTATTTCCGCCATCTC	NM 005573.3	63	1.9
*FDPS* *Farnesyldiphosphate synthase*	FDPS	TTGCTCCTCCCTCAGAATGAACGTGCCTCCAATGGCATTGTACTC	NM_001135821.1	59	1.8
*HDLBP* *High-density lipoprotein-binding protein*	HDLBP	GAACGCAGTTCACAGTACAG GTAACGGTGAAGGTCAAAGG	NM_005336	60	1.6
*HMGCR* *HMG-CoA Reduktase*	HMGCR	AAGTTTGCCCTCAGTTCC ACTGACATGCAGCCAAAG	NM 000859.2	59	1.9
*IGFBP3* *Insulin-like growth factor-binding protein 3*	IGFBP3	GCGCCAGGAAATGCTAGTGA GGGAATGTGTACACCCCTGG	NM_001013398.1	63	1.8
*LDLR* *Low-density lipoprotein receptor*	LDLR	CGACTGCAAGGACAAATCTG AGTCATATTCCCGGTACAC	NM_000527	60	1.6
*OPN* *Osteopontin*	OPN	TGATGACCATGTGGACAG ACCATTCAACTCCTCGCT	NM 000,900	63	1.8
*PCYOX* *Prenylcysteinoxidase 1*	PCYOX	CACTTCAGCAGCCTATTACC CCAGTTGCTCTCAAATACC	NM 016,297	59	1.9
*ZMPSTE24* *Zinc metalloprotease STE24*	ZMPSTE24	ACCACCGGAGTTAGGACAGA CCAGGGACTGAGTGATCTCAT	NM_005857.5	66	1.9
*GDF11* *Growth differentiation factor 11*	GDF11	AGGCCATTGGCAGAGCATCGAC GTCCCAGCCGAAAGCCTCAAAG	NM_005811.3	63	2.0

^1^ Accession numbers from reference sequences taken from GenBank are shown.
